# Inhibition of arginase by CB-1158 blocks myeloid cell-mediated immune suppression in the tumor microenvironment

**DOI:** 10.1186/s40425-017-0308-4

**Published:** 2017-12-19

**Authors:** Susanne M. Steggerda, Mark K. Bennett, Jason Chen, Ethan Emberley, Tony Huang, Julie R. Janes, Weiqun Li, Andrew L. MacKinnon, Amani Makkouk, Gisele Marguier, Peter J. Murray, Silinda Neou, Alison Pan, Francesco Parlati, Mirna L. M. Rodriguez, Lee-Ann Van de Velde, Tracy Wang, Melissa Works, Jing Zhang, Winter Zhang, Matthew I. Gross

**Affiliations:** 1 Calithera Biosciences, 343 Oyster Point Boulevard, Suite 200, South San Francisco, CA 94080 USA; 2 Max Planck Institute for Biochemistry, Martinsried, Germany; 3 Departments of Infectious Diseases and Immunology, St. Jude Children’s Research Hospital, Memphis, TN USA

**Keywords:** Arg1, Arg2, Arginase, Arginine, Checkpoint blockade, Granulocyte, Immunotherapy, Myeloid derived suppressor cell, Tumor associated macrophage, Tumor microenvironment

## Abstract

**Background:**

Myeloid cells are an abundant leukocyte in many types of tumors and contribute to immune evasion. Expression of the enzyme arginase 1 (Arg1) is a defining feature of immunosuppressive myeloid cells and leads to depletion of L-arginine, a nutrient required for T cell and natural killer (NK) cell proliferation. Here we use CB-1158, a potent and orally-bioavailable small-molecule inhibitor of arginase, to investigate the role of Arg1 in regulating anti-tumor immunity.

**Methods:**

CB-1158 was tested for the ability to block myeloid cell-mediated inhibition of T cell proliferation in vitro, and for tumor growth inhibition in syngeneic mouse models of cancer as a single agent and in combination with other therapies. Tumors from animals treated with CB-1158 were profiled for changes in immune cell subsets, expression of immune-related genes, and cytokines. Human tumor tissue microarrays were probed for Arg1 expression by immunohistochemistry and immunofluorescence. Cancer patient plasma samples were assessed for Arg1 protein and L-arginine by ELISA and mass spectrometry, respectively.

**Results:**

CB-1158 blocked myeloid cell-mediated suppression of T cell proliferation in vitro and reduced tumor growth in multiple mouse models of cancer, as a single agent and in combination with checkpoint blockade, adoptive T cell therapy, adoptive NK cell therapy, and the chemotherapy agent gemcitabine. Profiling of the tumor microenvironment revealed that CB-1158 increased tumor-infiltrating CD8^+^ T cells and NK cells, inflammatory cytokines, and expression of interferon-inducible genes. Patient tumor samples from multiple histologies expressed an abundance of tumor-infiltrating Arg1^+^ myeloid cells. Plasma samples from cancer patients exhibited elevated Arg1 and reduced L-arginine compared to healthy volunteers.

**Conclusions:**

These results demonstrate that Arg1 is a key mediator of immune suppression and that inhibiting Arg1 with CB-1158 shifts the immune landscape toward a pro-inflammatory environment, blunting myeloid cell-mediated immune evasion and reducing tumor growth. Furthermore, our results suggest that arginase blockade by CB-1158 may be an effective therapy in multiple types of cancer and combining CB-1158 with standard-of-care chemotherapy or other immunotherapies may yield improved clinical responses.

## Background

Myeloid cells in the tumor microenvironment (TME) are associated with poor prognosis across multiple types of cancer, including lung, colorectal, and breast [1]. Tumor-infiltrating myeloid cells contribute to an immunosuppressive TME through multiple mechanisms, constraining anti-tumor immunity and hindering immunotherapy [2, 3]. Agents that aim to block myeloid cell-mediated immunosuppression are currently in pre-clinical and clinical development, however there are no approved therapies specifically directed against tumor-associated myeloid cells [4, 5].

The major populations of tumor infiltrating myeloid cells include tumor-associated macrophages (TAMs), myeloid-derived suppressor cells (MDSCs), and granulocytes [2, 6, 7]. A feature common to all of these immunosuppressive cells is their expression of the enzyme arginase 1 (Arg1) [8]. Arg1 catalyzes hydrolysis of the amino acid L-arginine to produce urea and L-ornithine, thereby depleting extracellular L-arginine [9]. T cells are auxotrophic for L-arginine, requiring the amino acid for the rapid and successive rounds of proliferation that follow T cell antigen receptor (TCR)-dependent activation of effector cells [10–12]. In some inflammatory settings, myeloid-mediated arginine depletion is essential for suppressing excessive T cell proliferation [13]. Blocking Arg1 activity in the context of cancer could therefore shift the balance of L-arginine metabolism to favor lymphocyte proliferation. Indeed, in murine studies, injection of the arginase inhibitor nor-NOHA or genetic disruption of *Arg1* in the myeloid compartment resulted in reduced tumor growth, indicating that Arg1 is pro-tumorigenic [14, 15]. Thus, pharmacological inhibition of Arg1 is a compelling therapeutic strategy for the treatment of cancer.

Here we describe CB-1158, a potent and orally-bioavailable small-molecule inhibitor of Arg1. In T cell co-cultures, CB-1158 reversed myeloid cell-mediated immunosuppression and restored T cell proliferation. In murine syngeneic tumor models, CB-1158 shifted the tumor immune landscape toward a pro-inflammatory TME, resulting in tumor growth inhibition. CB-1158 augmented the efficacy of other anti-cancer agents, including gemcitabine, antibodies to immune checkpoints, adoptive T cell therapy, and adoptive NK cell therapy, to inhibit tumor growth. The therapeutic potential of targeting Arg1 was further supported in a screen of cancer patient samples that revealed an abundance of Arg1-expressing myeloid cells in tumors and high amounts of Arg1 in plasma. CB-1158 is currently in clinical trials for patients with solid tumor malignancies (NCT02903914).

## Methods

### Chemical compounds

CB-1158 was synthesized at Calithera Biosciences [16] and dissolved in 100% DMSO for biochemical assays or in Milli-Q water (Millipore, Billerica, MA) for cell-based assays and in vivo studies. No endotoxin contamination of CB-1158 preparations was observed. All other chemicals were purchased from Sigma (St. Louis, MO) unless indicated otherwise.

### Flow cytometry antibodies

The following anti-mouse antibodies were used for flow cytometry: CD45-V450 (30F11), CD45-BV510 (30F11), CD45-BV605 (30F11), CD8-BV510 (53–6.7), CD25-BV421 (PC61), CD25-BV605 (PC61) from BD Biosciences (San Jose, CA); CD3-PerCP-eFluor710 (17A2), CD45-PE-Cy7 (30F11), NKp46-eFluor660 (29A1.4), CD11b-PE-Cy7 (M1/70), CD68-PE-Cy7 (FA-11) from eBioscience (Thermo Fisher Scientific, Waltham, MA); CD3-PE (17A2); CD68-BV421 (FA-11), CD206-AlexaFluor488 (C068C2), CD11b-PerCP-Cy5.5 (M1/70), CD11b-BV605 (M1/70) from BioLegend (San Diego, CA); CD11b-PE (M1/70) from Stemcell Technologies (Vancouver, Canada); and Arg1-APC (polyclonal) from R&D Systems (Minneapolis, MN). The following anti-human antibodies were used for flow cytometry: CD66b-PE (G10F5), CD4-PerCP-Cy5.5 (SK3), CD8-APC (RPA-T8) from BD Biosciences; and CD15-eF450 (HI98) from eBioscience.

### Recombinant arginase activity assays

Recombinant full-length human Arg1 was purchased from Enzo Life Sciences (Farmingdale, NY). Recombinant human arginase 2 (Arg2) comprising amino acids 23–254 was purchased from US Biological (Salem, MA). Activity assays using 2 nM Arg1 or 4 nM Arg2 were performed in reaction buffer (137 mM NaCl, 2.7 mM KCl, 8 mM Na_2_HPO_4_, 2 mM KH_2_PO_4_, 0.005% Triton X-100, 0.5 mM DTT, 0.5 mM MgCl_2_, 0.1 mM CaCl_2_, and 160 μM or 20 mM L-arginine, pH 7.4) at 37 °C for 30 min with a dose-titration of CB-1158. Activity was determined by a spectrophotometric assay using the QuantiChrom Urea Assay Kit (BioAssay Systems, Hayward, CA) or by quantification of the generation of ^13^C(5)-L-ornithine from ^13^C(6)-L-arginine using a SCIEX API4000 mass spectrometer (Applied Biosystems, Foster City, CA). Urea produced or ^13^C(5)-L-ornithine peak areas were plotted and fitted to a four-parameter equation using GraphPad Prism software (San Diego, CA) to determine IC_50_ values.

### Native arginase activity in cell lysates

Human granulocytes or erythrocytes were purified from healthy donor peripheral blood using a pan-granulocyte negative selection kit (Stemcell Technologies) or centrifugation on a Ficoll layer, respectively. Frozen human hepatocytes were purchased from XenoTech (Kansas City, KS). Lysates were prepared by microtip sonication followed with clarification by centrifugation. Plasma samples from renal cell carcinoma (RCC) patients were obtained by Ficoll centrifugation of whole blood purchased from Conversant Biologics (Huntsville, AL). Granulocyte lysate was assayed at 0.094 mg/mL, as determined by bicinchoninic acid/BCA protein assay (ThermoFisher), in reaction buffer. Erythrocyte or hepatocyte lysates were assayed at concentrations empirically determined to consume 10–15% of ^13^C(6)-L-arginine in 30 min at 37 °C. Arginase activity was determined in lysates and plasma by quantification of the generation of ^13^C(5)-L-ornithine from ^13^C(6)-L-arginine in the presence of a dose-titration of CB-1158.

### Native arginase activity in intact cells

Intracellular arginase activity was determined for the arginase-expressing HepG2 and K-562 cell lines as follows. HepG2 cells were seeded at 100,000 cells per well one day prior to treatment with CB-1158. K-562 cells were seeded at 200,000 cells per well on the day of CB-1158 treatment. Cells were treated with a dose-titration of CB-1158 in SILAC RPMI-1640 media (Life Technologies/Thermo Fisher Scientific) containing 5% heat-inactivated and dialyzed FBS, antibiotics/anti-mycotic, 10 mM L-arginine, 0.27 mM L-lysine, and 2 mM L-glutamine. The medium was harvested after 24 h and urea generated was determined with the QuantiChrom Urea Assay Kit. Wells containing media without cells were used as background controls. For assessing the effect of CB-1158 on Arg1 in primary hepatocytes, frozen human hepatocytes (XenoTech) were thawed, allowed to adhere onto collagen-coated wells for 4 h, and then incubated for 48 h in SILAC-RPMI containing 10 mM L-ornithine, no L-arginine, and a dose-titration of CB-1158, at which time the media were analyzed for urea.

### Nitric oxide (NO) synthase (NOS) activity assays

Activity of 50 μM CB-1158 against 3 NOS isoforms, recombinant murine inducible NOS, recombinant bovine endothelial NOS, and native rat cerebellar neuronal NOS, was determined at Eurofins/Cerep Panlabs (Taipei, Taiwan) by either quantitation of radiolabeled L-citrulline or spectrophotometric measurement of nitrite.

### Cell culture

All cell culture reagents were purchased from Corning (Corning, NY) unless indicated otherwise. The human cell lines, HepG2 and K-562, and the murine cell lines, LLC1 (LLC), B16-F10 (B16), CT26.WT (CT26), and 4T1 were obtained from American Type Culture Collection (ATCC, Manassas, VA). HepG2, K-562, CT26, and 4T1 were maintained in RPMI-1640 (Corning). B16 was maintained in DMEM (Corning). LLC was maintained in DMEM (ATCC). All media were supplemented with 10% fetal bovine serum (FBS), plus penicillin, streptomycin, and amphotericin. Cell lines were grown at 37 °C in a humidified 5% CO_2_ atmosphere.

### Cytotoxicity assays

Cells were seeded in fully-supplemented RPMI-1640 medium, treated with a dose-titration of CB-1158 in triplicate wells, and incubated for 72 h. Cytotoxicity was assayed by the addition of CellTiterGlo reagent according to the manufacturer’s instructions (Promega, Madison, WI) followed by fluorescence quantification on a Molecular Devices plate reader (Sunnyvale, CA).

### T cell and NK cell proliferation assays

T cells or NK cells were purified from healthy donor human blood or from murine splenocytes using a negative selection kit for the appropriate cell type and species from Stemcell Technologies. Isolated T cells or NK cells were loaded with carboxyfluorescein succinimidyl ester (CFSE, Thermo Fisher) and stimulated for 72–96 h in complete growth medium containing a minimum of either 50 μM L-arginine (NK cells) or 100 μM L-arginine (T cells). For T cell stimulation, a solution of 10 μg/mL anti-CD3 (human clones UCHT1 or OKT3; murine clone 145-2C11) was used to coat the wells of a 96-well plate and then T cells were stimulated on immobilized anti-CD3 in the presence of 2 μg/mL soluble anti-CD28 (human clone CD28.2; murine clone 37.51). NK cells were stimulated with recombinant IL-2. Proliferation was quantified by analyzing CFSE dilution by flow cytometry (Guava flow cytometer, Millipore, Billerica, MA or Attune NxT flow cytometer, ThermoFisher).

### T cell/myeloid cell co-culture assays

Granulocytes were purified from healthy donor peripheral blood using a pan-granulocyte negative selection kit (Stemcell Technologies) and incubated in SILAC-RPMI medium containing 10% charcoal-stripped FBS, antibiotics/anti-mycotic, 0.27 mM L-lysine, 20 μM MnCl_2_, 100 μM L-arginine, pH 7.4, and a dose-titration of CB-1158. Freshly isolated granulocytes were incubated for 48 h at 37 °C, during which time they spontaneously activate as determined by increased surface expression of CD66b and scatter properties. T cells isolated from the same healthy donor using a pan-T cell isolation kit (Stemcell Technologies) were loaded with CFSE and plated with immobilized anti-CD3 and soluble anti-CD28 in the presence of the aged granulocytes. The cells were co-cultured at several ratios of granulocytes to T cells as indicated in the figure or at a fixed ratio of 4 T cells to 1 granulocyte. Co-cultures were incubated for 3–4 days, at which time the medium was analyzed for L-arginine and L-ornithine by mass spectrometry and T cell proliferation was determined by flow cytometry. Granulocytic MDSC (G-MDSC) or granulocytes from cancer patients were isolated from whole blood purchased from Conversant Biologics. G-MDSCs were purified from the PBMC layer of a Ficoll gradient by positive selection for CD66b^+^ cells. Granulocytes were purified from the RBC layer of a Ficoll gradient using Hetasep (Stemcell Technologies). Granulocytes and G-MDSCs were characterized by flow cytometry for CD66b expression. Freshly isolated G-MDSC or granulocytes were incubated in co-culture medium containing 100 μM L-arginine for 48 h, at which time the cells were removed and the G-MDSC- or granulocyte-conditioned media were used for incubating healthy donor CFSE-loaded T cells on immobilized anti-CD3/soluble anti-CD28 for 3–4 days. Cytokines were quantified in the media from T cell co-culture assays using the Cytometric Bead Array kit according to the manufacturer’s instructions (BD Biosciences).

### Murine tumor studies

Female wild-type C57BL/6 and Balb/c mice (5–6 weeks old) were purchased from Charles River Laboratories (Hollister, CA). Severe combined immune deficient (SCID, B6.CB17-PrkdcSCID/SzJ) and Pmel-1 TCR transgenic (B6.Cg-Thy1a/Cy Tg(TcraTcrb)8Rest/J) mice (5–6 weeks old) were purchased from The Jackson Laboratory (Bar Harbor, ME). All mice were housed and treated in accordance with Institutional Animal Care and Use Committee guidelines. For the 4T1 tumor model, 10^5^ cells were injected orthotopically into the mammary fat pad; for all other tumor models, 10^6^ cells were injected subcutaneously (s.c.) in the right flank. For all studies, CB-1158 was administered by oral gavage twice per day at 100 mg/kg starting on study day 1 (1 day after tumor implant). Control groups received vehicle (water) twice daily by gavage. Tumor volume measured by digital caliper (length × width × width/2) and body weight were recorded three times per week. Animals were euthanized when tumors necrotized or volumes reached 2000 mm^3^. For the CT26 model, anti-PD-L1 antibody (5 mg/kg, clone 10F.9G2, BioXCell, West Lebanon, NH) was injected intraperitoneally (i.p.) on days 5, 7, 9, 11, 13, and 15. For the 4T1 model, anti-CTLA-4 antibody (5 mg/kg, clone 9H10, BioXCell) was injected i.p. on days 2, 5, and 8; anti-PD-1 antibody (5 mg/kg, clone RMP1–14, BioXCell) was injected i.p. on days 3, 6, and 9. 4T1 tumors were harvested on study day 25 into Fekete’s solution and tumor nodules were enumerated visually. Gemcitabine (Selleckchem, Houston, TX) was dosed 50 mg/kg i.p. on days 10 and 16 for the CT26 model, 60 mg/kg i.p. on days 6 and 10 for the LLC model, or 30 mg/kg i.p. on day 5 for the 4T1 model [17, 18]. With these regimens, gemcitabine modestly reduces tumor growth and spares most tumor-infiltrating immune cells, allowing for the evaluation of combination activity with CB-1158. For CD8^+^ cell depletion, mice were injected i.p. with anti-CD8 antibody (25 mg/kg, clone 2.43, BioXCell) on days −1, 0, + 5, and +10. For NK cell depletion, mice were injected i.p. with anti-NK1.1 antibody (25 mg/kg, clone PK136, BioXCell) in the LLC and B16 models or with anti-Asialo GM1 sera (20 μL, Wako Chemicals, Richmond, VA) in the CT26 model, per the same schedule as anti-CD8.

### Conditional *Arg1* deleted mice

*Arg1* floxed mice were crossed to the *Tie2-Cre* deleter strain (The Jackson Laboratory) as previously described [19]. Experimental mice were generated from crossing *Arg1* ^*Flox/Flox*^ *; Tie2-Cre* ^*+*^ males with *Arg1* ^*Flox/Flox*^ *; Tie2-Cre* ^*−*^ females, with *Cre* negative littermates serving as wild-type controls. Mice were housed and treated in accordance with protocols approved by the Institutional Animal Care and Use Committee at St. Jude Children’s Research Hospital. LLC cells (10^6^ per mouse) were injected s.c. in the flank region. Mice were orally gavaged with either 100 mg/kg of CB-1158 or an equivalent volume of vehicle control (water) every 12 h for 14 days. Mice were euthanized, and tumors excised and weights recorded. Myeloid deletion of *Arg1* was confirmed via western blotting of IL-4-stimulated bone marrow-derived macrophages for all animals.

### Adoptive T cell transfer studies

Activated gp100-specific CD8^+^ (Pmel-1) T cells were generated as described in Ya et al. [20]. Briefly, splenocytes from Pmel-1 TCR transgenic mice were isolated, pulsed with 1 μM of murine gp100_25–33_ (Anaspec, Fremont, CA) and expanded for 1 week in the presence of 60 IU/mL recombinant human IL-2 (Peprotech, Rocky Hill, NJ). Cells were >90% CD8^+^ V_β_13^+^ T cells as determined by flow cytometry. C57BL/6 mice were inoculated s.c. with B16 tumor cells. CB-1158 was administered by oral gavage twice per day at 100 mg/kg starting 1 day after tumor implant. On day 7, lymphopenia was induced by a non-myeloablative chemotherapy regimen of 250 mg/kg cyclophosphamide and 50 mg/kg fludarabine administered i.p. The chemotherapy regimen was administered to all groups. On day 9, mice were administered 1 × 10^6^ Pmel-1 T cells intravenously (i.v.). Mice receiving Pmel-1 T cells also received recombinant human IL-2 (200,000 IU/dose) administered i.p. twice daily for 3 days starting the day of T cell transfer.

### Adoptive NK cell transfer studies

Balb/c mice were inoculated i.v. with 10^5^ CT26 cells. On the same day as tumor inoculation, 10^6^ NK cells (isolated from Balb/c spleens the day before injection and incubated with recombinant IL-2 and IL-15 for 18 h) were transferred to the mice. The injected NK cells were profiled by flow cytometry to be CD25^+^ and 80–90% pure with less than 0.4% T cells. Mice were treated with vehicle or CB-1158 for 14 days and then lungs were harvested into Fekete’s solution and tumor nodules enumerated visually.

### Tumor dissociation and flow cytometry

Tumor-bearing mice treated with vehicle or CB-1158 (100 mg/kg BID) were sacrificed for flow cytometry analysis on study day 9 (B16), day 10 (4T1), or day 14 (CT26 and LLC). Excised tumors were placed on ice in RPMI-1640 medium containing 5% FBS, minced with a razor blade, and dissociated in RPMI-1640 supplemented with mouse tumor dissociation enzymes (Miltenyi Biotec, Bergisch Gladbach, Germany) on a GentleMACS Octo Dissociator With Heat (Miltenyi Biotec) according to the manufacturer’s instructions. Dissociated tumors were strained through 70 μm nylon mesh, washed with cold PBS containing 2% FBS, blocked with anti-CD16/CD32 (Fc block antibody, eBioscience), and stained for cell surface antigens. For B16 and 4T1 tumors, washed dissociated tumor cells were incubated with Dead Cell Removal MicroBeads (Miltenyi Biotec) and applied to a magnetic column prior to staining. For intracellular staining, cells were fixed and permeabilized using buffers purchased from R&D Systems or eBioscience for cytoplasmic or nuclear antigens, respectively. All tumor flow experiments were acquired on an Attune NxT flow cytometer and analyzed with FlowJo software version 10 (Ashland, OR), using fluorescence-minus-one controls for gating and single-stained OneComp eBeads (eBioscience) to set compensation matrices.

### Gene expression analysis

LLC tumors from mice (*N* = 6 per group) treated with vehicle or CB-1158 (100 mg/kg twice daily) for 13 days were collected, placed into neutral buffered formalin overnight, transferred into 70% ethanol, and shipped to Core Diagnostics (Hayward, CA) for paraffin embedding. RNA was extracted for gene expression analysis and transcripts were quantified by NanoString Technologies (Seattle, WA).

### Cytokine analysis

LLC tumors from mice (*N* = 5 per group) treated with vehicle or CB-1158 (200 mg/kg twice daily) for 14 days were collected and flash frozen in liquid nitrogen. Tumors were homogenized in 50 mM Tris-HCl buffer containing 2 mM EDTA, pH 7.4 and protease inhibitors. The homogenate was centrifuged and the supernatant was collected and re-frozen. Cytokines in the supernatant were quantified by Myriad Rules Based Medicine (Austin, TX).

### Immunohistochemistry (IHC)

Automated IHC was performed by Indivumed (Hamburg, Germany) using the Discovery XT staining platform (Roche Diagnostics/Ventana Medical Systems, Mountain View, CA) on formalin-fixed and paraffin-embedded (FFPE) samples and tumor tissue microarrays (TMA). The rabbit anti-human Arg1 monoclonal antibody clone EPR6672(B) from Abcam/Epitomics (Burlingame, CA) was validated using 8 different cases of hepatocellular carcinoma (HCC) and one sample of normal liver tissue as positive control tissue; normal tonsil tissue and isotype control antibody were used as negative controls. IHC was performed on 11 different tumor histologies: non-small cell lung cancer (NSCLC, squamous and adenocarcinoma), breast cancer (triple negative and non-triple negative), gastric adenocarcinoma, colorectal cancer (CRC), prostate adenocarcinoma, pancreatic cancer, ovarian cancer, bladder cancer, and RCC. Arg1^+^ cells per mm^2^ were quantified by digital histopathology (OracleBio, Scotland, UK).

### Multiparameter immunofluorescence

Tumor TMAs containing samples from patients with lung squamous cell carcinoma, CRC, RCC, esophageal carcinoma, and head and neck cancer were purchased from US Biomax and US Biolabs (Rockville, MD). Multiparameter immunofluorescence using the MultiOmyx platform for markers including Arg1, CD15, and CD68 was performed and analyzed by GE Clarient/NeoGenomics Laboratories (Aliso Viejo, CA).

### Plasma Arg1 and L-arginine

The amount of Arg1 protein in plasma samples was determined by enzyme linked immunosorbent assay (ELISA, BioVendor, Asheville, NC) in specimens from healthy volunteers and patients with head and neck cancer (*N* = 5), HCC (*N* = 3), mesothelioma (*N* = 3), CRC (*N* = 3), T cell prolymphocytic leukemia (*N* = 2), melanoma (*N* = 2), bladder cancer (*N* = 4), NSCLC (*N* = 11), small cell lung cancer (*N* = 17), undefined lung cancer (*N* = 6), acute myeloid leukemia (*N* = 9), RCC (*N* = 9), and breast cancer (*N* = 2). Plasma L-arginine was determined by mass spectrometry in samples from patients with mesothelioma (*N* = 3), CRC (N = 3), NSCLC (*N* = 9), small cell lung cancer (*N* = 3), undefined lung cancer (*N* = 3), head and neck cancer (*N* = 3), and T cell prolymphocytic leukemia (*N* = 2). All cancer patient samples were purchased from Conversant Biologics.

## Results

CB-1158 was tested in biochemical and cellular assays for the ability to inhibit arginase enzymes from a variety of sources. CB-1158 inhibited recombinant human Arg1 (IC_50_ = 86 nM) and the related enzyme Arg2 (IC_50_ = 296 nM) (Table 1). Arg2 catalyzes an identical chemical reaction and shares 60% sequence identity with Arg1 and differs in its tissue distribution and subcellular localization [21]. A second enzyme class that also metabolizes L-arginine and is implicated in inflammation is NOS, which produces L-citrulline and the biological mediator NO [8]. CB-1158 was tested for the ability to inhibit the three NOS isoforms, endothelial NOS, neuronal NOS, and inducible NOS. No inhibition of NOS enzymes was observed in the presence of 50 μM CB-1158 (Table 1). These results show CB-1158 is a potent inhibitor of arginase with no activity against NOS.

**Table 1:** Biochemical potency of CB-1158 against arginase and NOS activity

	CB-1158, IC_50_ (*nM*)
Purified enzyme assay	
Recombinant human Arg1	86 (±25)
Recombinant human Arg2	296 (±5)
Recombinant bovine endothelial NOS	N/A
Rat cerebellar neuronal NOS	N/A
Recombinant murine inducible NOS	N/A
Cell lysate assay	
Human granulocyte lysate	178 (±28)
Human erythrocyte lysate	116
Human hepatocyte lysate	158 (±23)
Cancer patient plasma	122 (±32)

Mean IC_50_ values in nanomolar for CB-1158 inhibition of purified recombinant arginases or native arginases in cell lysates or cancer patient plasma. Standard deviations are indicated in parentheses. Recombinant Arg1 assays (*N* = 3) were performed in duplicate wells in the presence of 160 μM L-arginine. Recombinant Arg2 assays (*N* = 2) were performed in triplicate wells with 20 mM L-arginine. Arginase activity assays using human granulocyte lysate (*N* = 3), human erythrocyte lysate (*N* = 1), human hepatocyte lysate (*N* = 4), and cancer patient plasma (*N* = 5) were performed in duplicate wells with 160 μM L-arginine. Purified NOS enzyme activity was assayed in the presence 50 μM CB-1158, which showed no inhibitory activity against the three NOS isoforms

*N/A* not applicable

CB-1158 was next tested for the ability to inhibit native Arg1 in lysates of human granulocytes, peripheral blood erythrocytes, and primary hepatocytes [22–24]. We found that CB-1158 inhibited native arginase in lysates with similar potency to that observed for recombinant arginases (Table 1). In addition, Arg1 protein and activity have been reported to be elevated in the plasma of cancer patients compared to healthy donors [25, 26] and we observed inhibition of arginase activity by CB-1158 in plasma isolated from cancer patients (Table 1).

Granulocytic Arg1 is inactive until it is exocytosed [10, 23, 27], however active Arg1 is localized in the cytoplasm in other cell types, including liver hepatocytes. CB-1158 was next tested for the ability to inhibit endogenous arginase in intact cells. CB-1158 exhibited low potency against intracellular arginase in the hepatocellular carcinoma (HCC) cell line HepG2, the chronic myelogenous leukemia cell line K-562, and primary human hepatocytes (Table 2). The low potency of CB-1158 against arginase in intact cells is likely due to inefficient penetration of CB-1158 across the cell membrane. These results show that CB-1158 is a potent and specific inhibitor of extracellular arginase.

**Table 2:** Potency of CB-1158 on arginase activity in intact cells

Intact cell assay	CB-1158, IC_50_ (*μM*)
Human HepG2 cell line	32 (±5.6)
Human K562 cell line	139 (±8.8)
Primary Human Hepatocytes	210

Mean IC_50_ values in micromolar for CB-1158 inhibition of arginase activity in intact cells. Standard deviations are indicated in parentheses. HepG2 (*N* = 3) and K562 (*N* = 2) cell lines were plated in duplicate wells in the presence of 10 mM L-arginine. For primary human hepatocytes (*N* = 1), arginase activity was measured in duplicate wells in the presence of media containing 10 mM L-ornithine and lacking L-arginine. Arginase activity was measured as production of urea in the media after 24 h

**Fig. 1:**
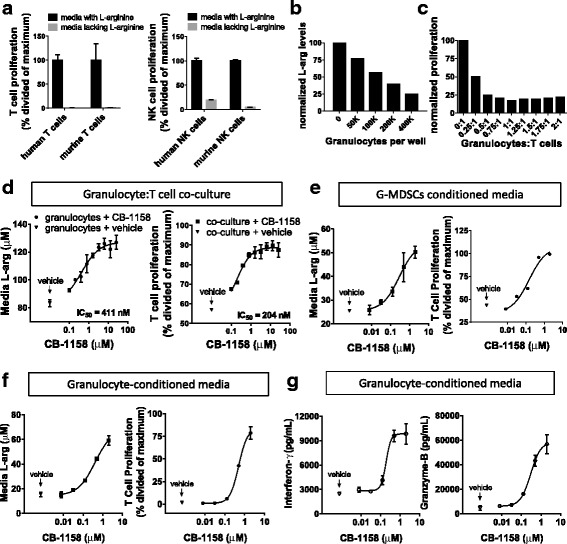
Inhibition of arginase reverses myeloid cell-mediated suppression of in vitro T cell proliferation. **a** T cells *(left)* and NK cells *(right)* require extracellular L-arginine for proliferation. CFSE-loaded T cells or NK cells were stimulated with anti-CD3/anti-CD28 or IL-2, respectively, in media either containing or lacking L-arginine. Proliferation was measured after 72 h by flow cytometry. **b** Isolated human granulocytes deplete L-arginine from the media, measured after 48 h by LC/MS. **c** Human peripheral blood T cells are suppressed from anti-CD3/anti-CD28-induced proliferation by co-culture with granulocytes isolated from the same healthy donor. **d** *left*, CB-1158 inhibits the consumption of arginine from the media by granulocytes in a dose-dependent manner; *right*, CB-1158 inhibits granulocyte-mediated suppression of T-cell proliferation in a dose-dependent manner. The ratio of granulocytes to T cells in the co-cultures was 0.25 to 1. **e**, CB-1158 reverses T cell suppression conferred by granulocytic MDSCs. Media conditioned by granulocytic-MDSCs purified from a lung cancer patient’s blood inhibited T-cell proliferation and is depleted of L-arginine, and both effects are reversed in a dose-dependent manner by CB-1158. *Left*, arginine amounts in the media; *right*, T-cell proliferation. The ratio of MDSCs conditioning the media to T cells was 1 to 1. **f**, Conditioned media from purified granulocytes isolated from a head and neck cancer patient’s blood inhibited T-cell proliferation and are depleted of L-arginine, and both effects are reversed in a dose-dependent manner by CB-1158. *Left*, arginine amounts in the media; *right*, T-cell proliferation. The ratio of granulocytes conditioning the media to T cells was 0.5 to 1. **g**, CB-1158 reverses the inhibition of secretion of interferon-γ and granzyme-B conferred by cancer patient granulocytes. Media from panel (*f)* were analyzed by Cytometric Bead Array

To determine if CB-1158 can restore lymphocyte proliferation in the context of immunosuppressive arginase-expressing myeloid cells, we first confirmed that lymphocytes require exogenous L-arginine to proliferate. Purified T cells or NK cells were stimulated with anti-CD3/anti-CD28 or IL-2, respectively, in the presence or absence of L-arginine in the media. Proliferation of human and murine T cells (Fig. 1a, left) and NK cells (Fig. 1a, right) only occurred in media that contained L-arginine, as expected [10, 11, 28, 29].

To determine if arginase activity is necessary for myeloid cell-mediated suppression of T cell proliferation, T cell proliferation was assayed in co-culture with human myeloid cells in the presence or absence of CB-1158. Granulocytes are an abundant myeloid cell recruited from peripheral blood to sites of wound healing, infection [30], and the TME [25, 31]. Arg1 is stored in cytoplasmic granules and upon activation, which occurs spontaneously in vitro, granulocytes release active Arg1 into the extracellular milieu [10]. Purified human granulocytes from a healthy donor consumed L-arginine from the media (Fig. 1b). When activated granulocytes were co-cultured with autologous T cells, T cell proliferation was inhibited (Fig. 1c). The addition of CB-1158 blocked depletion of L-arginine from the media (Fig. 1d, left) and restored T cell proliferation to 90% of the proliferation observed for T cells without granulocytes (Fig. 1d, right). These results show that L-arginine depletion by Arg1 is necessary for granulocyte-mediated suppression of T cell proliferation in vitro, and that CB-1158 reverses this immunosuppression.

We next tested if CB-1158 could block T cell suppression conferred by myeloid cells derived from cancer patients. G-MDSC purified from the peripheral blood of a patient with lung cancer or granulocytes purified from a head and neck cancer patient were cultured for 48 h and the conditioned media were then used in T cell proliferation assays. T cells from healthy donors were used in lieu of the cancer patient donors’ T cells due to the limited amount of sample available from individual patients. Cancer patient-derived G-MDSCs or granulocytes reduced the amount of L-arginine in the media, and arginine depletion was blocked by CB-1158 (Fig. 1e and f, left panels). T cell proliferation was inhibited in the media conditioned by G-MDSC or granulocytes, and proliferation was restored to 99% or 79%, respectively, of control levels by the addition of CB-1158 (Fig. 1e and f, right panels). CB-1158 also restored secretion into the media of the T cell inflammatory cytokines interferon-γ and granzyme-B (Fig. 1g). Together these data demonstrate that inhibition of arginase by CB-1158 blocks myeloid cell-mediated immunosuppression, rescuing T cell proliferation and cytokine secretion.

**Fig. 2:**
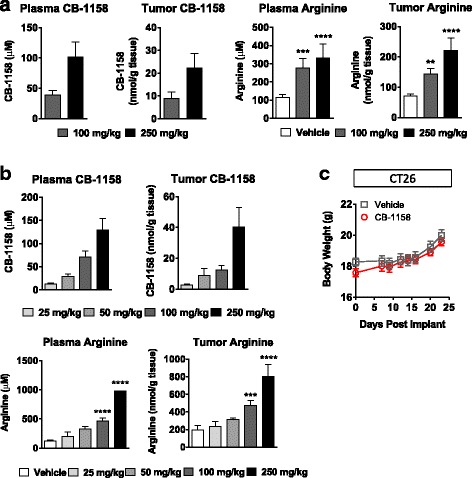
CB-1158 has favorable pharmacokinetic and pharmacodynamic properties in vivo with no overt signs of toxicity. LLC tumor-bearing mice (*N* = 5 per group) were administered a single dose of CB-1158 (**a**) or 5 twice-daily doses (**b**) and samples were collected 2 h after the last dose. CB-1158 and L-arginine in plasma and tumor lysates were measured by LC/MS. **c** Body weights of mice inoculated with CT26 cells and dosed with vehicle or CB-1158 twice daily for 23 days. (**** *P* < 0.0001; *** *P* < 0.001; ** *P* < 0.01 vs. vehicle)

To determine if arginase inhibition by CB-1158 could be tested for anti-tumor efficacy in mouse models of cancer, pharmacokinetic and pharmacodynamic studies were performed in tumor-bearing mice. Following a single dose of CB-1158 or BID dosing for 5 days, dose-dependent exposure of CB-1158 was observed in both plasma and tumors (Fig. 2a and b). Oral dosing of CB-1158 in tumor-bearing mice also raised the amount of L-arginine in plasma and tumors, indicating an on-target pharmacodynamic effect of CB-1158 (Figs. 2a and b). Importantly, CB-1158 was well-tolerated at doses of 100 mg/kg twice daily for 23 days, with no significant clinical observations or impact on body weight (Fig. 2c).

**Fig. 3:**
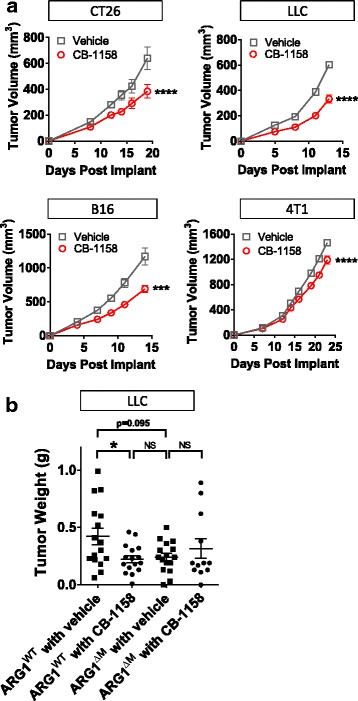
Arginase inhibition reduces tumor growth in vivo. **a** CB-1158, dosed at 100 mg/kg twice daily, reduced tumor growth as a single agent in multiple syngeneic mouse models of cancer (*N* = 10 per group). **b** Mice lacking *Arg1* gene expression in the myeloid compartment (indicated as ARG1^ΔM^) grow smaller tumors than mice containing wild-type *Arg1* (indicated as ARG1^WT^), and treatment of ARG1^ΔM^ mice with CB-1158 does not further reduce tumor growth, indicating on-target activity of CB-1158 (*N* = 16 per group). (T test: **** *P* < 0.0001; * *P* < 0.05)

CB-1158 was tested in multiple syngeneic murine models of cancer. Blocking arginase with CB-1158 significantly inhibited the growth of CT26, LLC, B16, and 4T1 tumors (Fig. 3a). Confirmation that CB-1158 targets Arg1 in vivo was assessed with a genetically altered mouse strain containing a conditional disruption of *Arg1* in the myeloid lineage [14, 19, 32]. LLC cells injected into *Arg* ^*flox/flox*^ *; Tie2-Cre* ^*+*^ mice (Fig. 3b, indicated as ARG1^ΔM^) grew smaller tumors that were similar in size to CB-1158-treated *Arg* ^*flox/flox*^ *; Tie2-Cre* ^*−*^ mice (Fig. 3b, indicated as ARG1^WT^), and CB-1158 treatment of *Arg* ^*flox/flox*^ *; Tie2-Cre* ^*+*^ animals conferred no further reduction in tumor growth, consistent with specific on-target inhibition of Arg1 by CB-1158 (Fig. 3b). Together, these results provide evidence that Arg1 activity promotes tumor growth and that elimination of myeloid cell *Arg1* expression or pharmacological blockade of arginase by CB-1158 limits tumor growth in vivo.

**Fig. 4:**
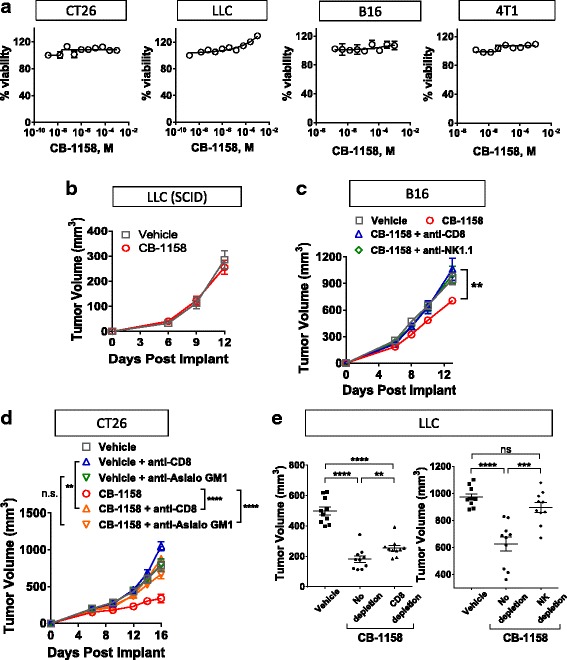
CB-1158 requires an intact immune system for efficacy. **a** CellTiterGlo assays (72 h) were performed on the indicated cell lines with a dose-titration of CB-1158. **b** B6.CB17-Prkdc (SCID)/SzJ mice were implanted with LLC cells and CB-1158 was dosed 100 mg/kg twice daily. **c-e** Tumor growth inhibition by CB-1158 in the B16 (**c**), CT26 (**d**), and LLC (**e**) models is mediated by CD8^+^ and NK cells. Tumor-bearing mice were treated with depleting antibodies and dosed twice-daily with vehicle or 100 mg/kg CB-1158. Tumors from LLC studies were analyzed on study day 13 (CD8^+^ cell depletion) or study day 14 (NK cell depletion). (**** *P* < 0.0001; *** *P* < 0.001; ** *P* < 0.01)

A series of experiments was performed to address the in vivo mechanism of action of CB-1158. First, we confirmed that CB-1158 is not directly cytotoxic to murine cancer cell lines by assaying the growth of CT26, LLC, B16, and 4T1 cell lines in the presence of a dose-titration of CB-1158. No growth inhibition of murine cancer cell lines was observed in the presence of 1 mM CB-1158 (Fig. 4a). Next, to test if the mechanism of in vivo efficacy of CB-1158 is immune cell-mediated, CB-1158 was administered to LLC tumor-bearing SCID mice. The efficacy of CB-1158 was abrogated in the SCID background (Fig. 4b), indicating CB-1158 requires an intact immune system to inhibit tumor growth. To confirm the immune cell-mediated mechanism of action of CB-1158, tumor growth was assessed in mice lacking immune cell subsets. Depletion of either CD8^+^ cells or NK cells in the B16 (Fig. 4c) and CT26 (Fig. 4d) tumor models blocked the efficacy of CB-1158, indicating that both CD8^+^ cells and NK cells are required for the full anti-tumor effect of CB-1158 in these models. In the LLC tumor model, depletion of NK cells resulted in loss of efficacy, while depletion of CD8^+^ cells had a smaller effect (Fig. 4e). Collectively, these results indicate that the anti-tumor effect of CB-1158 is mediated by the immune system and requires cytotoxic lymphocytes.

**Fig. 5:**
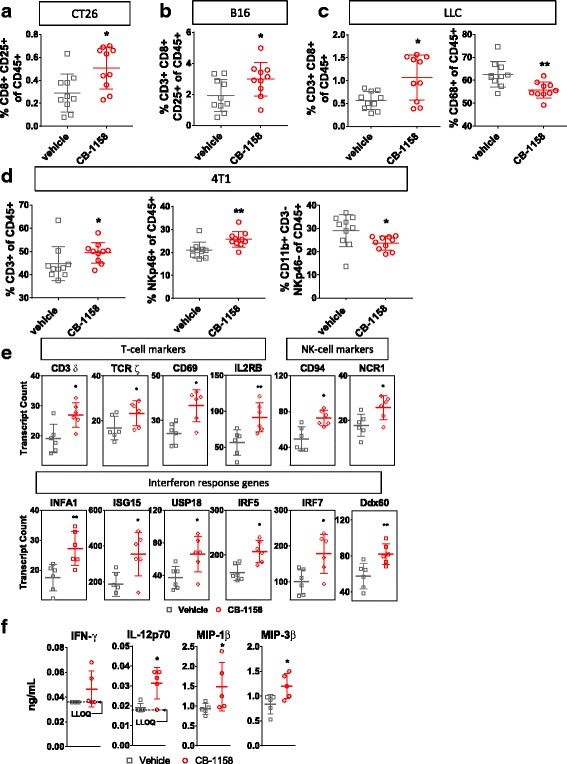
CB-1158-treated animals have increased tumor-infiltrating cytotoxic cells and decreased myeloid cells. **a** CT26 tumors from animals treated with CB-1158 had increased CD8^+^ CD25^+^ T cells as determined by flow cytometry compared to vehicle-treated animals on study day 14 (*N* = 10 per group). **b** In the B16 model, CB-1158 treatment resulted in an increase in CD25^+^ CD8^+^ T cells observed on study day 9 (*N* = 10 per group). **c** In the LLC model, CB-1158 treatment resulted in increased tumor infiltrating CD8^+^ T cells and decreased CD68^+^ myeloid cells observed on study day 14 (*N* = 10 per group). **d** In the 4T1 model, CB-1158 treatment resulted in increases in both T cells and NK cells and a decrease in myeloid cells observed on study day 10 (*N* = 10 per group). **e** CB-1158 increases T cell and NK cell markers and interferon response genes. mRNA transcripts in LLC tumors from mice treated with vehicle or 100 mg/kg CB-1158 twice daily were determined by Nanostring (*N* = 6 per group). **f** Cytokines and chemokines in LLC tumors from mice treated with vehicle or 200 mg/kg CB-1158 twice daily were determined by Luminex (*N* = 5 per group). (T test: ** *P* < 0.01; * *P* < 0.05)

To further investigate the immune cell-mediated mechanism of action of CB-1158, flow cytometry was performed on tumors from animals treated with vehicle or CB-1158 and changes in specific immune cell populations were quantified. In each of the models where tumor growth was inhibited by CB-1158, we observed statistically significant changes in immune cell populations indicative of an increase in inflammation in the TME (Fig. 5). In the CT26 (Fig. 5a) and B16 (Fig. 5b) models, CB-1158 treatment resulted in an increase in tumor-infiltrating activated CD8^+^ CD25^+^ cytotoxic T cells compared to vehicle-treated controls. In the LLC model, CB-1158 treatment caused an increase in CD8^+^ T cells in the tumor, as well as a decrease in CD68^+^ macrophages, compared to vehicle-treated controls (Fig. 5c). In the 4T1 model, where CB-1158 had a modest effect on tumor growth, we nevertheless observed changes in immune cell populations consistent with increased inflammation in the tumor: an increase in CD3^+^ T cells, an increase in NK cells, and a decrease in myeloid cells in the tumors of CB-1158-treated animals compared to vehicle-treated controls (Fig. 5d). Consistent with increased inflammation in the tumors of CB-1158-treated animals compared to vehicle controls, CB-1158 was linked to increases in transcripts of interferon-responsive genes (Fig. 5e) and increases in inflammatory cytokines (Fig. 5f). Taken together, our data indicate that the molecular mechanism of tumor growth inhibition by CB-1158 is immune cell-mediated and results from an increase in inflammation in the tumor microenvironment that includes an increase in cytotoxic lymphocytes and a decrease in myeloid cells.

**Fig. 6:**
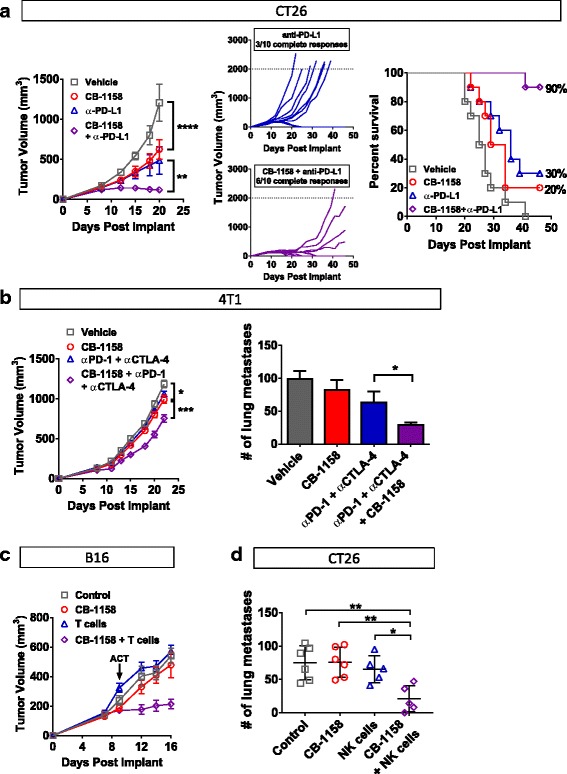
CB-1158 combines with immune checkpoint blockade, T cell transfer, or NK cell transfer to inhibit tumor growth. **a** CB-1158 in combination with PD-L1 blockade inhibited tumor growth in the CT26 model. Growth curves *(left and center)* and *s*urvival curves *(right)* are shown*.* *N* = 10 per group. **b** CB-1158 combined with CTLA-4 and PD-1 blockade to inhibit tumor growth and lung metastases in the 4T1 tumor model. *N* = 10 per group. **c** CB-1158 and adoptive T cell transfer inhibited tumor growth in the B16 model. Non-myeloablative chemotherapy regimen of cyclophosphamide plus fludarabine (C/F) was administered to all groups, and IL-2 was dosed to groups receiving T cells. On study day 9, Pmel-1 T cells were transferred to mice in the T cell groups (ACT). *N* = 10 per group. **d** CB-1158 and adoptive NK cell transfer reduced lung metastases in the CT26 model. *N* = 6 control; *N* = 7 CB-1158; *N* = 5 NK cells; *N* = 5 CB-1158 + NK cells. (T test: **** *P* < 0.0001; *** *P* < 0.001; ** *P* < 0.01; * *P* < 0.05)

In growing tumors, effective immunity may be blocked by more than one suppressive mechanism, including expression of immune checkpoint proteins and depletion of essential nutrients from the TME [33–35]. We reasoned that combining CB-1158 with other immune-modulating agents might further reduce tumor growth and tested this idea with four different combinations. First, CT26 tumor-bearing mice were treated with CB-1158 in combination with the checkpoint blockade therapy anti-PD-L1. While tumor growth was reduced with CB-1158 or anti-PD-L1 as monotherapies, tumor growth inhibition was enhanced by combining the two agents (Fig. 6a, left). Complete tumor regression was achieved in three out of ten mice treated with anti-PD-L1 alone, whereas the combination therapy resulted in six complete responses (Fig. 6a, center), with 90% survival at study day 46 for the combination group compared to 30% survival for the anti-PD-L1 single agent group (Fig. 6a, right). Combination efficacy with checkpoint blockade therapy was further explored in the orthotopic 4T1 tumor model, where the addition of CB-1158 to the doublet regimen of anti-CTLA-4 and anti-PD-1 resulted in a significant reduction in growth of the primary tumor as well as a decrease in the number of lung metastases (Fig. 6b). Next, we used two different cellular immunotherapies in combination with CB-1158. B16 tumor-bearing mice treated with CB-1158 in combination with T cells specific for the PMEL tumor antigen exhibited significantly reduced tumor growth compared to mice treated with either single agent (Fig. 6c). Of note, the significant tumor growth inhibition in mice treated with the combination of CB-1158 plus T cells is consistent with the striking survival benefit that was reported for T cell therapy in mice deleted for myeloid *Arg1* [32]. We also used NK cell therapy in a lung metastasis model. CT26 tumor-bearing mice treated with CB-1158 in combination with NK cells had significantly fewer lung metastases compared to control groups (Fig. 6d). These results indicate that CB-1158 may be an attractive combination agent with other immuno therapies.

**Fig. 7:**
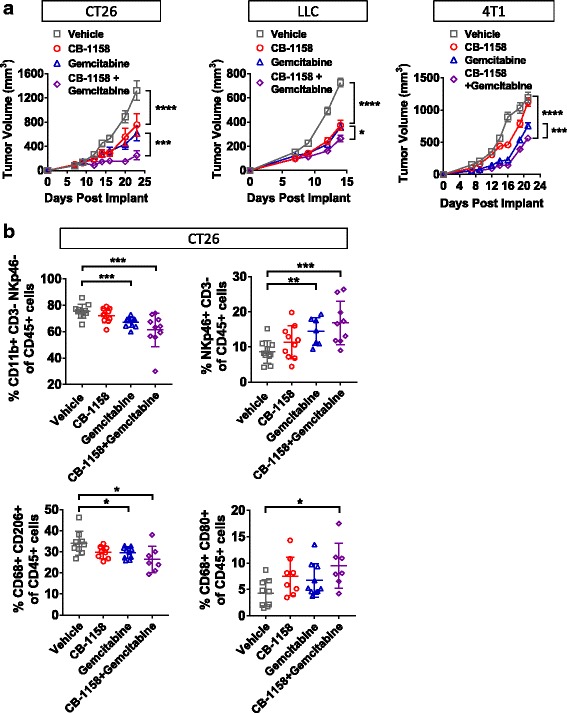
CB-1158 combines with gemcitabine to inhibit tumor growth. **a** CB-1158 in combination with gemcitabine treatment in the CT26 model *(left),* the LLC model *(center),* or the 4T1 model *(right)* inhibited tumor growth. N = 10 per group. **b** On study day 14, CT26 tumors from animals treated with CB-1158 and gemcitabine had decreased myeloid cells *(upper left)*, increased NK cells *(upper right)*, decreased suppressive macrophages *(lower left)*, and increased inflammatory macrophages *(lower right)* as determined by flow cytometry compared to vehicle-treated animals

Some standard-of-care chemotherapeutics have been observed to modulate the immune infiltrate [36, 37]. While the primary mechanism of action of gemcitabine, a nucleoside analog, is considered to be inhibition of DNA synthesis, it has also been observed to reduce MDSCs [38–40]. Therefore, we reasoned that gemcitabine may augment the activity of CB-1158. Indeed, CB-1158 treatment in combination with gemcitabine resulted in a significant increase in tumor growth inhibition in the CT26, LLC, and 4T1 tumor models (Fig. 7a). We investigated the mechanism of action of the combination of CB-1158 and gemcitabine treatment in the CT26 tumor model by performing flow cytometry on these tumors. Gemcitabine treatment resulted in a significant decrease in CD11b^+^ myeloid cells in the tumors (Fig. 7b). We note that although gemcitabine can be toxic to immune cells, both lymphocytes and myeloid cells remained present in gemcitabine-treated animals with the dose regimen that was used. Interestingly, NK cells and inflammatory (M1-type, CD80^+^) macrophages were significantly increased, while immunosuppressive (M2-type, CD206^+^) macrophages were significantly reduced in tumors of animals treated with the combination of CB-1158 and gemcitabine, indicating an increase in inflammation and a decrease in immune suppression in the tumor microenvironment (Fig. 7b).

**Fig. 8:**
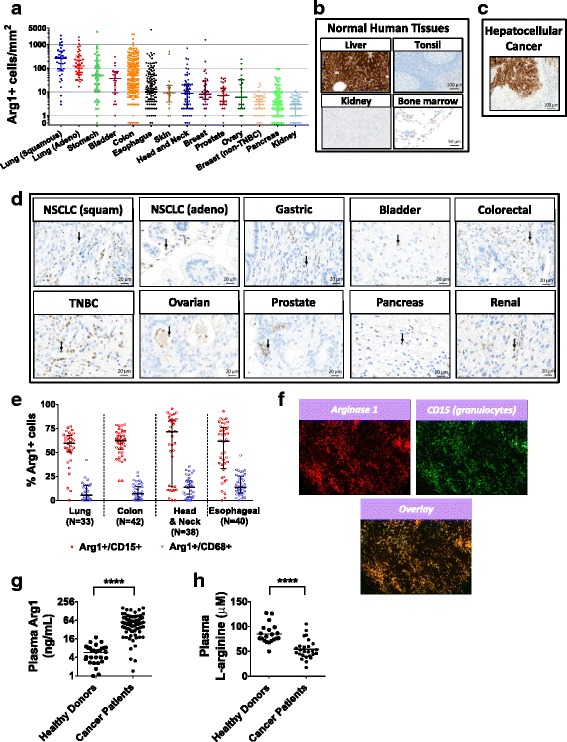
Arginase 1 is abundant in multiple types of cancer. **a** Immunohistochemistry of human tumor tissue microarrays stained with an anti-Arg1 antibody were quantified for Arg1-positive infiltrating granulocytes by digital histopathology. **b-d** Immunohistochemistry staining for Arg1 in sections of normal human tissues (*N* = 33 tissues analyzed) and human tumor tissues (*N* = 12 tumor histologies analyzed). Representative images are shown. Red arrows point to arginase-expressing myeloid cells. **e** Percentage of Arg1-positive cells that co-express the granulocyte marker, CD15, or macrophage marker, CD68, in tumor tissue microarrays as determined by quantitation of MultiOmyx immunofluorescence. **f** Immunofluorescent staining (MultiOmyx) of a tumor section from a patient with head & neck cancer shows numerous Arg1-positive granulocytes. **g** Plasma Arg1 protein determined by ELISA from cancer patients (*N* = 76 from 13 different histologies) and healthy volunteers (*N* = 31). **h** Plasma L-arginine determined by LC/MS from cancer patients (*N* = 26 from 7 different histologies) and healthy volunteers (*N* = 20). (**** *P* < 0.0001 vs. healthy donor)

To investigate which tumor types may be more likely to respond to arginase inhibition, we interrogated human tumor microarrays by immunohistochemistry for Arg1 protein expression. The Arg1 antibody was validated using normal liver tissue as a positive control and normal tonsil tissue as a negative control (Fig. 8b). We found an abundance of Arg1^+^ infiltrating immune cells in multiple tumor types, with especially high numbers in tumors of the lung, gastrointestinal tract, and bladder (Fig. 8a and d). Tumor cells were largely negative for Arg1 staining with the exception of HCC (Fig. 8c). Tumor tissue microarrays were also stained by multi-parameter immunofluorescence for Arg1 and other immune cell markers, and we found that Arg1 was more frequently associated with the granulocytic marker CD15 than with the macrophage marker CD68 (Fig. 8e), and in some cases, striking co-localization between Arg1 and CD15 was observed (Fig. 8f). These data confirm Arg1 expression in multiple tumor types.

In addition to tumor expression, Arg1 protein and activity have been observed in the peripheral blood and have been reported to be higher in the plasma of some cancer patients compared to healthy volunteers [25]. To investigate whether the amount of Arg1 is higher in cancer patients than in healthy donors, Arg1 protein was measured in the plasma of 31 healthy donors and 76 cancer patients across 13 different histologies (see Methods). Arg1 was significantly higher for cancer patients compared to the healthy volunteers (Fig. 8g). Peripheral blood L-arginine has also been reported to be lower in cancer patients [25, 26]. Plasma L-arginine was measured for 20 healthy volunteers and 26 cancer patients across 7 different histologies. L-arginine was significantly lower for the cancer patients compared to the healthy individuals (Fig. 8h). These results suggest that cancer patients may experience immune suppression that is associated with higher circulating Arg1 and lower amounts of L-arginine compared to healthy individuals, and that inhibiting circulating Arg1 and raising plasma L-arginine with CB-1158 could confer an immune benefit in the context of cancer.

## Discussion

We reasoned that raising arginine levels would be immune-stimulatory in the context of cancer for the following reasons: first, cytotoxic lymphocytes require exogenous arginine for proliferation in response to in vitro stimulation [10, 11, 28, 29]; second, many cancer patients are immunosuppressed and have lower plasma arginine compared to healthy individuals [25, 26]; and lastly, activated immunosuppressive myeloid cells consume arginine and compete with other arginine auxotrophs such as cytotoxic lymphocytes in the TME for this amino acid [8, 12]. Thus, raising arginine in cancer patients could be critical for the immune system to mount an effective anti-tumor response.

Arginine depletion by myeloid cells is primarily mediated by the enzymes Arg1 and NOS [8]. While there is conflicting data regarding the role of NOS in inflammation [32, 41], Arg1 activity in myeloid cells has been shown to be immunosuppressive and pro-tumorigenic: first, Arg1-expressing myeloid cells consume arginine from the media and suppress T cell activity in co-culture, and importantly, T cell proliferation can be restored either by restoring arginine to the media or by the addition of an arginase inhibitor, showing that arginase activity is necessary for the observed immune suppression (Fig. 1) [10, 11, 25, 42, 43]. Secondly, genetic ablation of *Arg1* in the myeloid compartment of tumor-bearing mice was shown to reduce tumor growth, indicative of a pro-tumorigenic and immune suppressive role for Arg1 in vivo [13, 14, 19, 32]. Thirdly, blocking arginase activity pharmacologically with nor-NOHA [15] or CB-1158 (this report) reduces tumor growth. The present study additionally demonstrates that Arg1 inhibition with CB-1158 raises tumor and plasma arginine and increases inflammation in the TME. Together these data argue that arginase activity is immunosuppressive and provide validation for arginase as a cancer immunotherapy drug target. A second arginase isoform, Arg2 is a constitutively-expressed mitochondrial matrix protein found at low levels in many tissues and at high levels in kidney and intestine [21]. Since Arg2 activity also affects plasma L-arginine levels, Arg2 could also be a regulator of immune function. In support of this notion, Arg2 has been reported to promote maternal-fetal immune tolerance [44].

A potential concern with systemic arginase inhibition is interrupting its function within the urea cycle, an important pathway in the liver for detoxifying ammonia generated from the breakdown of amino acids. In spite of this concern, pharmacological arginase inhibition has been well-tolerated in several animal studies, including one study involving a rat model of hypertension in which nor-NOHA was injected over a period of 10 weeks [45], as well as multi-day studies of nor-NOHA in mice [15]. In addition, we have observed that twice-daily oral dosing of CB-1158 was well-tolerated in mice for at least 40 days. Lack of apparent hepatic toxicity may be explained by several observations. First, CB-1158 does not readily enter cells, exhibiting IC_50_ values for intracellular arginase in the HepG2 and K562 cell lines that are two orders of magnitude higher than for soluble arginases in cell lysates (Tables 1 and 2). Secondly, the subcellular localization and regulation of urea cycle Arg1 may protect it from pharmacological inhibition. In hepatocytes, urea cycle Arg1 is tightly associated at the mitochondria in a multi-enzyme complex, and studies using semi-permeabilized cells and radiolabeled substrate have demonstrated that tight channeling of product and substrate occurs among successive enzymes of the urea cycle: argininosuccinate synthase, argininosuccinate lyase, and arginase [46]. Thus, hepatic Arg1 may be less accessible to CB-1158 compared to cytoplasmic or extracellular arginase in plasma, tumors, and inflamed tissues. When we tested CB-1158 for the ability to inhibit arginase in intact primary hepatocytes (Table 2), we used media containing ornithine, not arginine, and to our knowledge, urea generated under these conditions should require a complete urea cycle. Therefore, this assay may be a measure of the Arg1 activity that is exclusively associated with the urea cycle. However we note that the low potency of CB-1158 on primary hepatocytes could also be due to the inability of CB-1158 to penetrate the cell membrane.

Arginine supplementation has been investigated as a potential therapy for cancer patients and the clinical results suggest that raising arginine levels may be beneficial. In one study of 18 colorectal cancer patients undergoing tumor resection, histopathologic analysis of biopsies revealed that supplementation of arginine prior to surgery resulted in an increase in CD16^+^ and CD56^+^ NK cells infiltrating the tumors [47]. In another study of 96 breast cancer patients, a significant increase in pathological response was observed in patients with small tumors receiving arginine compared to placebo [48]. These data indicate that supplemental arginine may have an immune-stimulatory and anti-tumor effect in cancer patients, and suggest that raising systemic arginine by pharmacological arginase inhibition would be similarly beneficial. However, major limitations to therapeutic oral arginine supplementation include severe gastrointestinal distress and extensive metabolism of arginine by the intestinal mucosa [9, 49–51]. CB-1158 treatment has the potential to both augment and maintain arginine levels in patients thereby avoiding the gastrointestinal distress and arginine oscillations inherent to dietary arginine supplementation.

Antibodies to T cell checkpoint proteins CTLA-4, PD-1, and PD-L1 have resulted in durable clinical responses for some cancer patients, but many patients do not respond [52, 53], suggesting that overcoming additional immunosuppressive mechanisms will be necessary to reactivate anti-tumor immunity in resistant patients. The cellular and molecular basis of resistance to checkpoint blockade is an area of intense investigation. Biomarker studies examining responsive and resistant patients point to a suppressive TME as one possibility to explain resistance. Patients with a higher baseline level of T cells infiltrating the tumor (T cell-inflamed tumors) compared to non-responsive tumors (cold tumors) were more likely to respond to anti-CTLA-4 or anti-PD-1 therapies [54], and patients resistant to anti-PD-1 therapy had higher baseline levels of tumor-infiltrating MDSCs compared to responders [55]. Furthermore, pre-clinical studies have shown that the depletion of essential extracellular metabolites in the TME, such as glucose and amino acids, can block T cell effector function [56, 57]. Together, these results suggest that immunosuppressive myeloid cells and depletion of essential metabolites may give rise to cold tumors that are resistant to checkpoint blockade and therefore targeting these myeloid cells and metabolic regulatory pathways, in combination with checkpoint antibodies, could restore inflammation and increase patient response rates. In support of this hypothesis, epacadostat, which inhibits the enzyme IDO-1 and prevents depletion of the essential amino acid tryptophan from the TME, is exhibiting striking results in combination with anti-PD-1 therapy in patients with melanoma, lung cancer, RCC, or head and neck carcinoma [58–62], arguing that targeting T cell checkpoints and immunosuppression by the TME is an efficacious combination.

## Conclusions

In this report, we have shown that inhibition of arginase with CB-1158, a potent, selective, and orally-bioavailable small molecule, reverses suppression of T cell proliferation by blocking arginine depletion. CB-1158 reduced tumor growth in mouse models of cancer by increasing inflammation in the TME, confirming an immune-modulatory mechanism of action in vivo. In addition, CB-1158 treatment of tumor-bearing mice enhanced the efficacy of checkpoint blockade, adoptive T cell therapy, adoptive NK cell therapy, and gemcitabine treatment. A survey of human tumor microarrays and cancer patient plasma confirmed Arg1 expression is elevated in human cancer. These findings provide strong rationale for the clinical development of CB-1158 as an anti-cancer therapeutic agent. A Phase 1 study (NCT02903914) has been initiated to test the anti-tumor activity of CB-1158, as a monotherapy and in combination with anti-PD-1, in order to add to the armamentarium of agents that activate the immune system to fight cancer.

## Abbreviations

Arg1: Arginase 1
Arg2: Arginase 2
CFSE: Carboxy-fluorescein succinimidyl ester
CRC: Colorectal cancer
DC: Dendritic cell
ELISA: Enzyme-linked immunosorbent assay
FBS: Fetal bovine serum
FFPE: Formalin-fixed and paraffin-embedded
G-MDSC: Granulocytic myeloid-derived suppressor cell
HCC: Hepatocellular carcinoma
i.p.: Intraperitoneally
i.v.: Intravenously
IDO-1: Indoleamine-2,3-dioxygenase-1
IHC: Immunohistochemistry
MDSC: Myeloid-derived suppressor cell
NK: Natural killer
NO: Nitric oxide
NOS: Nitric oxide synthase
PD-L1: Programmed death ligand 1
RCC: Renal cell carcinoma
s.c.: Subcutaneously
SCID: Severe combined immune deficient
TAM: Tumor-associated macrophage
TCR: T cell antigen receptor
TMA: Tissue microarray
TME: Tumor microenvironment
